# Development of a novel ozone- and photo-stable HyPer5 red fluorescent dye for array CGH and microarray gene expression analysis with consistent performance irrespective of environmental conditions

**DOI:** 10.1186/1472-6750-8-86

**Published:** 2008-11-12

**Authors:** Mubasher Dar, Theresa Giesler, Rob Richardson, Christine Cai, Mike Cooper, Shahin Lavasani, Peter Kille, Thierry Voet, Joris Vermeesch

**Affiliations:** 1 GE Healthcare, 800 Centennial Ave, Piscataway, NJ 08855, USA; 2 Development, GE Healthcare, The Maynard Center, Cardiff, Wales, CF14 7YT, UK; 3 Cardiff School of Biosciences, Cardiff University, Museum Avenue, Cardiff, Wales, CF10 3TL, UK; 4 Department of Human Genetics, Katholieke Universiteit Leuven, Herestratt 49, 3000 Leuven, Belgium

## Abstract

**Background:**

Array-based comparative genomic hybridization (CGH) and gene expression profiling have become vital techniques for identifying molecular defects underlying genetic diseases. Regardless of the microarray platform, cyanine dyes (Cy3 and Cy5) are one of the most widely used fluorescent dye pairs for microarray analysis owing to their brightness and ease of incorporation, enabling high level of assay sensitivity. However, combining both dyes on arrays can become problematic during summer months when ozone levels rise to near 25 parts per billion (ppb). Under such conditions, Cy5 is known to rapidly degrade leading to loss of signal from either "homebrew" or commercial arrays. Cy5 can also suffer disproportionately from dye photobleaching resulting in distortion of (Cy5/Cy3) ratios used in copy number analysis. Our laboratory has been active in fluorescent dye research to find a suitable alternative to Cy5 that is stable to ozone and resistant to photo-bleaching. Here, we report on the development of such a dye, called HyPer5, and describe its' exceptional ozone and photostable properties on microarrays.

**Results:**

Our results show HyPer5 signal to be stable to high ozone levels. Repeated exposure of mouse arrays hybridized with HyPer5-labeled cDNA to 300 ppb ozone at 5, 10 and 15 minute intervals resulted in no signal loss from the dye. In comparison, Cy5 arrays showed a dramatic 80% decrease in total signal during the same interval. Photobleaching experiments show HyPer5 to be resistant to light induced damage with 3- fold improvement in dye stability over Cy5. In high resolution array CGH experiments, HyPer5 is demonstrated to detect chromosomal aberrations at loci 2p21-16.3 and 15q26.3-26.2 from three patient sample using bacterial artificial chromosome (BAC) arrays. The photostability of HyPer5 is further documented by repeat array scanning without loss of detection. Additionally, HyPer5 arrays are shown to preserve sensitivity and data quality from gene expression experiments.

**Conclusion:**

HyPer5 is a red fluorescent dye that behaves functionally similar to Cy5 except in stability to ozone and light. HyPer5 is demonstrated to be resistant to ozone at up to 300 ppb, levels significantly higher than commonly observed during summer months. Consequently, HyPer5 dye can be used in parallel with Cy3 under any environmental conditions in array experiments.

## Background

Cyanine family (Cy™3 and Cy5) of fluorescent dyes have been widely used in parallel microarray detection for array comparative genomic hybridization and gene expression analysis [1-3]. The large molar extinction coefficients and ease of enzymatic incorporation of Cy3 and Cy5 allows both dyes to be combined for high sensitivity detection of low copy targets even when sample amounts are limited [4,5]. However, a number of reports have been published documenting the instability of Cy5 dye to elevated ozone levels in the environment causing distortion of gene expression (Cy5/Cy3) ratios [6,7]. In summer months, when environmental ozone levels increase, microarray hybridization experiments can suffer disproportionately from Cy5 signal loss over time impacting quality of data acquired from arrays. Unlike Cy5, the signal and brightness from Cy3 remains stable during higher ozone periods. To circumvent Cy5 signal loss from ozone exposure, Branham et. al. have proposed an engineering solution based on installation of a carbon-filtration system to eliminate ozone inside laboratories [7]. While these systems can be effective in depleting ozone, they are expensive and difficult to engineer in open laboratory spaces allowing ozone levels to fluctuate posing continuous risk to data quality from often expensive arrays. Cy5 signal can also be impacted by dye photobleaching effects. Photobleaching can occur when arrays are exposed directly to light or when partially or even microscopically wet arrays are scanned for image acquisition. Like ozone, photobleaching of Cy5 leads to reduction of absolute signals obtained from the arrays.

To circumvent these problems, our laboratory has been active in fluorescent dye research to find a suitable alternative to Cy5 for microarray analysis with brightness and environmental stability matching Cy3. Here, we report on the development of such a novel red fluorescent dye, known as HyPer5, with high ozone- and photo-stability that yields reproducible microarray performance all year round – irrespective of environmental conditions.

In this article, we describe the properties of HyPer5 dye and compare its performance to Cy5. By using in-house fabricated arrays, we show resistance of HyPer5 signal to repeat exposures of ozone pulses of 300 ppb over time. Data is presented on the marked 3–4 fold improvement in the photostability of HyPer5 over Cy5 following exposure of dyes to incandescent light source. We functionally demonstrate the ability of HyPer5 to detect chromosomal aberrations at multiple loci in array CGH experiments using patient samples and ability to perform array rescanning without loss of resolution. Furthermore, gene expression analysis using mouse arrays is demonstrated to show equivalent performance between HyPer5 and Cy5 dye in message detection. Microarray labeling data is also presented showing HyPer5 incorporation rates matching Cy5.

## Results

**Figure 1:**
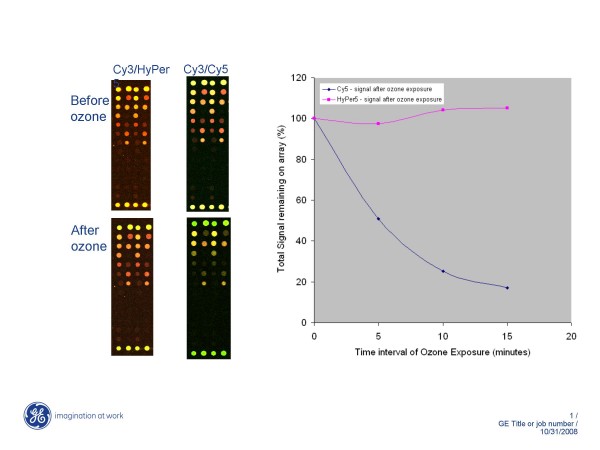
**a**. **Ozone treatments of HyPer5 and Cy5 arrays lead to only Cy5 signal loss.** These figures show sections of Cy3/HyPer5 and Cy3/Cy5 hybridized microarrays before (top) and after (bottom) 300 ppb ozone treatments. The ozone readings were monitored using a meter throughout the course of treatments. The first scan (no treatment) was set as time zero and the ozone images shown are following 10 minutes of total (two exposure of 5 minutes each) ozone exposure. Cy5 or HyPer5 signals appear as red spots and Cy3 signal as green spots. Near equal signal intensities of Cy3 and Cy5 or HyPer5 from individual features are observed as yellow spots. The signal from HyPer5 arrays remains unchanged following ozone treatments (compare top image to bottom). In contrast, sudden drop in Cy5 signal (red or yellow spots) is seen as conversion of yellow or red (top) features to green spots. **b**. Ozone stability of HyPer5 dye. Two separate in-house printed mouse arrays were hybridized with Cy3/Cy5 and Cy3/HyPer5 labelled cDNAs. Following initial scan (time zero), the arrays were exposed to 300 ppb of ozone for 5, 10, and 15 minute intervals. After each exposure, the slides were removed from the box and scanned. The slides were then immediately placed back inside the box again for longer exposures. The signal intensities were quantified and reported as the percentage of total Cy5 or HyPer5 signal following each ozone exposure relative to the total signal intensities at time zero. The data shows the sudden decline in Cy5 signal to 50% from 5 minutes of ozone exposure and drops to low level of 17% at 15 minute interval. In contrast, the HyPer5 signal remains completely intact following three 300 ppb ozone exposures demonstrating the resistance of dye signal to ozone in the chamber.

Seasonal effects on microarray data quality have identified ozone to be the root cause of Cy5 degradation. An increase in ozone levels to between 5–25 ppb are known to severely impact Cy5 signal [6]. We compared the ozone resistance of new HyPer5 against Cy5 by repeatedly exposing in-house fabricated and hybridized mouse arrays to short pulses of 300 ppb ozone over time (Figure 1a and 1b). For each exposure, the slides were placed inside a box along with the ozone generator. The 300 ppb ozone was delivered from an ozone generator for 5 minute intervals lasting up to 15 minutes (three treatments) with immediate scanning after each treatment. Since ozone tends to be very unstable, high (300 ppb) doses of gas were necessary for each treatment. As shown in Figure 1a (right images), a sudden reduction in Cy5 signal (red) was observed after two ozone treatments (10 minutes). Yellow features, corresponding to near equivalent Cy3 (green) and Cy5 (red) signal, now appear to have mostly green cast from disappearance of Cy5 signal. In contrast, HyPer5 signal was unchanged by ozone treatments. The individual features (red and yellow) did not change their colour after repeated ozone treatments indicating resistance of HyPer5 (and Cy3) to ozone (Figure 1a, left images).

Quantification of signals before and after ozone treatments showed an immediate and dramatic drop in Cy5 signal down to 50% within 5 minutes of exposure (Figure 1b). After 15 minutes, the detrimental effect of ozone on Cy5 resulted in greater than 80% signal loss. In contrast, HyPer5 signal was found to be completely intact following ozone treatments demonstrating the resistance of HyPer5 to ozone in the environment. Successive exposures of HyPer5 arrays to 300 ppb ozone for up to 15 minutes did not drop its signal. In fact, a slight increase in HyPer5 signal was noted after the third ozone exposure (15 minute time interval, see below). This data shows that HyPer5 is resistant to ozone and performs reliably on arrays even under elevated ozone conditions inside laboratories.

**Figure 2:**
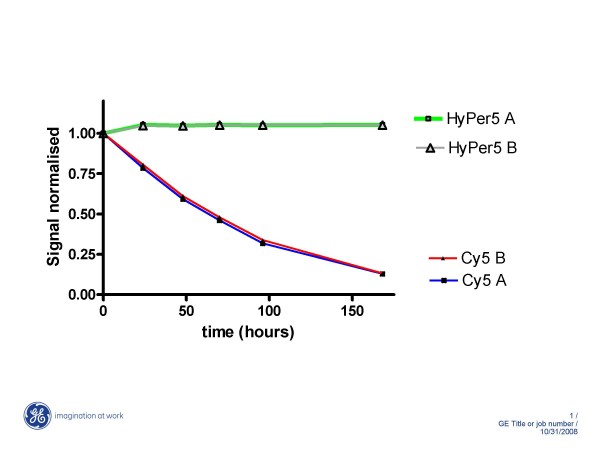
**Photo-stability of HyPer5 dye**. Normalized signals from two HyPer5 samples exposed to light source are shown in grey and green lines. The signals from matching Cy5 samples measured over time are shown in red and blue lines. The signals were normalized against control samples kept in the dark for both HyPer5 and Cy5 dyes. Greater than 3-fold difference in photostability was observed between HyPer5 and Cy5 over the tested interval.

Along with ozone sensitivity, Cy5 arrays can also suffer signal loss from dye photobleaching. The photobleaching properties of microarray dye can be characterized in two ways. First, one can measure the relative stability of dye from continuous exposure to a light source. Second, in a microarray experiment, dye photostability can be monitored by multiple rescanning of hybridized array to determine if any loss in signal or resolution is seen (see CGH rescanning data below). Both tests were used to measure the photobleaching property of HyPer5 against Cy5. In the first test, duplicate samples of both dyes were exposed to an incandescent light source for seven days, during that period their absorbance were measured at regular intervals (Figure 2). The data was normalized to control samples left in the dark for both dyes over the same time interval. As seen in Figure 2, both samples of HyPer5 dye were found to be completely photostable and resistant to photobleaching with no decline in absorbance readings over time. In contrast, Cy5 absorbance measurements showed steady decline from photobleaching with overall difference of more than 3 fold compared to HyPer5 data.

**Figure 3:**
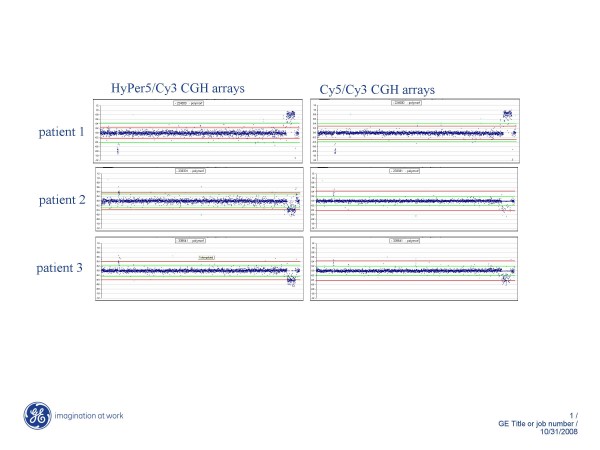
**Performance of HyPer5 dye in array CGH analysis.** Genomic DNA isolated from three patients were labelled separately with Cy5-dCTP and HyPer5-CTP using BioPrime Array CGH Genomic DNA Labeling system (Invitrogen, Carlsbad, CA). The control reactions were labelled with Cy3-dCTP. The HyPer5/Cy3 arrays are shown on the left and Cy5/Cy3 arrays on the right. Results from patient 1 are on top, patient 2 in the middle and patient 3 data are shown at the bottom arrays. Samples labelled with HyPer5-dCTP perform similarly to Cy5-labeled samples in 3K BAC CGH array as demonstrated by detection of aberrations at 2p21-16.3 and 15q26.3-26.2. The y-axis represents ratios of HyPer5/Cy3 or Cy5-Cy3 signal intensities in the logarithmic scale.

HyPer5 dye was tested functionally in array CGH assay by comparing its performance against Cy5. Genomic DNA isolated from three different patient samples (obtained from University Hospital Leuven) along with appropriate control samples were differentially labelled with Cy3/Cy5 and Cy3/HyPer5. The dye-labelled microarray targets were then hybridized onto commercially available 3K BAC arrays (Figure 3) and data analyzed for relative copy number changes using ratio analysis. As seen in Figure 3, HyPer5 dye labelled targets were able to detect known chromosomal aberration at 2p21-16.3 and 15q26.3-26.2 comparable to Cy5 targets, although initial signal intensity generated by the HyPer5 targets were slightly diminished. The lower brightness coefficient of HyPer5 relative to Cy5 (110,000 M^-1 ^vs. 250,000 M^-1^) contributes to observed lower signal intensity from HyPer5 array elements. HyPer5 dye was noticed to contribute slightly elevated noise to the data, although this had no impact on the ability to detect aberrations. The source of higher background is not known but may be attributed to the more hydrophilic nature of HyPer5 dye.

**Figure 4:**
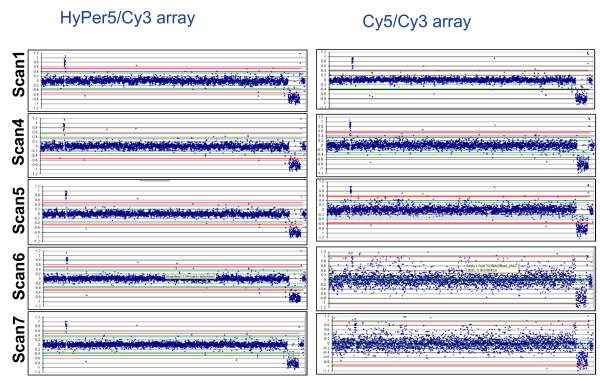
**Photostability of HyPer5 in array rescanning experiment.** The performance of HyPer5 to Cy5 is compared under similar environmental condition by rescanning BAC CGH array. Ozone readings were monitored to rule out degradation from ozone effects. HyPer5/Cy3 array data is shown on the left, whereas, Cy5/Cy3 arrays are depicted on the right. Rescanning results are shown from scan 1 (top arrays), scan 4, scan 5, and scan 7 (bottom arrays). Rescanning causes drop in Cy5 signal over time resulting in the inability of Cy5 arrays to detect aberration. In contrast, HyPer5 arrays can be rescanned multiple times without any loss of aberration detection.

As mentioned above, dye photostability can also be measured by array rescanning to determine if loss of signal or aberration detection is observed from repeat scanning. While Cy5 dye is highly bright, precautions have to be taken to prevent signal loss from light due to photobleaching particularly when imaging is performed using laser-based scanners [8]. We measured photo-stability of both dyes by the ability of arrays to be rescanned multiple times without suffering any distortion in dye ratios. Part of CGH arrays were rescanned multiple times over a period of 14 days (Figure 4). From the data, it is obvious that HyPer5 signals are as stable as Cy3 signals whereas Cy5 performance (signal) dropped quickly in a period of 5 to 8 days, resulting in loss of aberration detection and an increase in standard deviation (scatter).

Another important functional application of Cy3 and Cy5 lies in expression microarrays for analysis of gene expression [9]. Cy5 and HyPer5 dyes were compared side-by-side in gene expression analysis using in-house fabricated mouse arrays. Due to the lower extinction coefficient of HyPer5 (110,000 M^-1^), we found it necessary to use 30% more HyPer5 labelled cDNA onto each array for high sensitivity. Starting from mouse total RNA, HyPer5 was seen to perform similar to Cy5 and Cy3 dyes in parallel microarray hybridization experiments in the ability to detect similar number of genes from mouse array (Table 1).

**Table 1:** Hybridization data with HyPer5/Cy3 and Cy5/Cy3 dyes from mouse microarray printed with 3000 genes (in triplicates)

**Dye**	**Cy5 or HyPer5 mean signal**	**Cy3 mean signal**	**Count of genes above background**
HyPer5 array	2383	1847	4574

Cy5 array	2522	2292	4598

While HyPer5 dye is an ozone and photostable version of Cy5, we wanted to determine whether labelling efficiency of HyPer5 was comparable to Cy5. To test labelling, we synthesized HyPer5 both as N-hydroxy-succinimide (NHS) reactive dye and as deoxycytidine triphosphate nucleotide (HyPer5-dCTP) for microarray labelling experiments [10]. Starting from an input of 20 μg of mouse total RNA, we observed more than 2-fold higher labelling yield from HyPer5 using the NHS reactive dye (Table 2) with post-labelling method. With direct enzymatic (reverse transcriptase) incorporation reaction, HyPer5-dCTP was found to give equivalent target yield to Cy5-dCTP using conventional first-strand cDNA synthesis reactions (Table 2). The incorporation efficiency, as measured by nucleotide per dye ratio, of HyPer5 dye was seen to be comparable to Cy5. Higher labelling density was achieved with HyPer5 NHS, although HyPer5-dCTP incorporation was slightly lower than Cy5-dCTP.

**Table 2:** Labelled cDNA yield starting from 20 μg of mouse total RNA

	**HyPer5 NHS**	**Cy5 NHS**	**HyPer5-dCTP**	**Cy5-dCTP**
Dye incorporation	87.1	34.5	32	35

Nucleotide per dye ratio*	29	77	94	70

*Nucleotide per dye ratios was determined by [DNA yield × 1000]/[dye incorporation × 324.5]. Data represented is average yield from n = 35 for HyPer5 NHS, n = 33 for Cy5 = NHS, and triplicates for HyPer5- and Cy5-dCTP.

## Discussion

Cy5 is a bright fluor with high extinction coefficient. Being in the red region of the spectrum, it offers the advantage of performing microarray assays with little or no background effects. However, it is prone to oxidative damage caused by ozone in the environment. Here, we have described the properties of an alternative red dye, HyPer5 that is stable to ozone and light which can generate reliable microarray data. Microarray experiments performed with HyPer5 under elevated ozone conditions which rapidly degrade Cy5 show no drop in HyPer5 signal.

Typically microarray laboratories experience Cy5 signal loss during summer months when ozone levels rise to greater than 10 ppb. We have demonstrated HyPer5 performs functionally similar to Cy5 making HyPer5 a suitable replacement in microarray assays under challenging environmental conditions. Ozone experiments did show a modest increase in HyPer5 signal intensity after repeated exposures (see Figure 1). The cause of this increase is unknown although rescanning experiments using CGH arrays, where ozone levels were being monitored and remained low, also exhibited a similar modest increase in HyPer5 signal. In CGH experiments, the signal increase leads to improved detection of aberrations benefiting from slightly higher signal-to-noise values (compare the tighter scatter plot of scan 7 with plot in scan 1 of HyPer5/Cy3 CGH array in Figure 4). In an attempt to understand the maximal effect of HyPer5 signal increase, we accelerated this process by placing HyPer5 arrays in desiccator overnight followed by image acquisition and data analysis. These results showed an improvement in signal to noise in the range of 5–25% (data not shown). While this property of HyPer5 improves CGH data, it may introduce slight variability in ratio analysis from arrays rescanned multiple times or if arrays were delayed for several hours prior to scanning. However, the major property of HyPer5 in providing ozone stability far exceeds the modest rise in HyPer5 signal obtained from repeat scanning, ozone exposure, or slide storage. Furthermore, the slight increase in HyPer5 signal did not alter the ratios significantly to impact aberration detection in array CGH experiments.

Another difference between HyPer5 and Cy5 is exhibited in the photostability of new HyPer5 dye. Constant exposure of arrays to light during different steps of the microarray experiment can lower Cy5 signal intensities when arrays are finally imaged. There is also a slight but significant loss of Cy5 signal seen from repeat array scanning that is attributed to dye photobleaching. HyPer5 dye has been shown to be photostable and resistant to photobleaching. The main benefit of photostability lies in the ability to perform multiple rescanning experiments without suffering from loss in resolution on array CGH slides. Additionally, stability of HyPer5 signal may also allow the possibility of archiving Cy3 and HyPer5 arrays which were previously impossible due to Cy5 instability.

Functional data indicates that HyPer5 can be substituted for Cy5 in array analysis. The protocols used for hybridization essentially allow HyPer5 labelled samples to replace Cy5. Rather than making direct dye swap on arrays, we find it necessary to use ~30% more HyPer5 labelled targets for array hybridization than conventionally used with Cy5. This difference comes from the relatively lower brightness coefficient of HyPer5 to Cy5 which needs to be compensated by using more labelled targets for hybridization. When using HyPer5-dCTP for labelling, increasing the amount of dye in the reaction will increase yield of labelled targets desired for hybridization. For the HyPer5 NHS reactive dye, labelling conditions can be kept identical due to higher coupling efficiency of HyPer5 NHS compared to Cy5.

## Conclusion

The new HyPer5 dye is a more ozone- and photo-stable version of Cy5 for microarray applications. We have demonstrated HyPer5 to be resistant to repeated exposures of ozone at 300 ppb released from ozone generator inside a chamber, conditions at which Cy5 shows dramatic loss of signal. Parallel photobleaching experiments performed on HyPer5 and Cy5 show HyPer5 to be resistant to light-induced damage with 3-fold improvement in dye-stability over Cy5. The ozone- and photo-stability of HyPer5 makes this dye highly suitable for array applications particularly during periods of high ozone and humidity levels in summer months, conditions at which Cy5 data can be highly unpredictable. Additionally, the photostability of HyPer5 is particularly advantageous in allowing multiple rescanning of arrays without loss of detection. The functional use HyPer5 is demonstrated in array CGH and gene expression experiments to acquire microarray data for copy number analysis. The lower brightness coefficient of HyPer5 may dictate the use of more labelled targets for hybridization to maximize array sensitivity. With high labelling and coupling efficiency of HyPer5, it can be easily adapted to existing Cy5 protocols for both array CGH and microarray gene expression analysis to perform array experiments under any environmental conditions.

## Methods

### Microarray dye labelling and cDNA synthesis

To determine the labelling efficiency, cDNA synthesis was performed from mouse total RNA (20 μg) in the presence of 1 μl of HyPer5- or Cy5-dCTP according to the protocol outlined in the CyScribe™ First-strand Labelling kit (GE Healthcare, Piscataway, NJ) [11]. Labelling efficiency for HyPer5 NHS and Cy5 NHS was measured following cDNA synthesis and post-labelling using the CyScribe Post labelling kit (GE Healthcare, Piscataway, NJ) starting from 20 μg of mouse total RNA. For array CGH analysis, the labelling efficiencies of HyPer5-, Cy5- and Cy3-dCTP were tested using the BioPrime™ Array CGH Genomic Labelling System (Invitrogen, Carlsbad, CA). All experiments were started from 150 ng of human genomic DNA that was amplified overnight in a 25 μl reaction using 0.5 μl of Klenow DNA polymerase. The labelling yield and efficiency of dye incorporation was quantified by spectroscopy at wavelengths of 260 nm, 550, and 650 nm for DNA, Cy3, and Cy5/HyPer5, respectively. For labelled probe yield determination, an extinction coefficient of 110, 000 M^-1 ^was used for HyPer5 and 250,000 M^-1^,150,000 M^-1 ^for Cy5 and Cy3, respectively.

### Physical properties and availability of HyPer5 dye

HyPer5 is a red fluorescent dye with a slight (~14 nm) red-shift compared to Cy5. HyPer5 has sharp absorbance and emission spectra with maxima at wavelengths of 664 nm and 680 nm, respectively (Cy5 values are 650 nm and 670 nm, respectively). The quantum yield of HyPer5 (0.3) is almost identical to Cy5 (0.28) [12]. Brightness (extinction) coefficient of HyPer5 is 110, 000 M^-1^cm^-1 ^compared to 250,000 for Cy5 M^-1^cm^-1^. The HyPer5 dye is commercially available from GE Healthcare and is supplied in two conventional formats, HyPer5-dCTP (product code 28923183) and HyPer5-NHS reactive dye (product code 28922418), for various labelling techniques. The core structure of HyPer5 is different from cyanine dye and hence recommended labelling protocols outlined above and available with product should be followed.

### Array fabrication and hybridization

Purified amplicons (~200 ug/ml) representing a full length mouse cDNAs together with appropriate controls, prelabelled landmarks and print buffer negative controls were added to an equal volume of DMSO and 10 ul transferred to 384 well plates. These composite plates were then used as source plates for printing an array of 48 (4 × 12) patches incorporating 336 spots (21 × 16) onto a Ultra-GAP glass slides (Corning) using split SMP3 pins (Telecham) mounted in a Spotarray 72 (Perkin-Elmer). This printing regime yield spots of approximately 120 μm in diameter. Reporters were cross-linked to the surface by baking at 80°C for 2 hours, and UV cross linking. Slides were stored in the dark and under a vacuum until required.

Array hybridization were performed using 50 pmoles of HyPer5 labelled mouse cDNA or 35 pmoles of Cy5, or Cy3 labelled cDNA either manually or on automated hybridization station (Lucidea SlidePro, GE Healthcare) in microarray hybridization buffer (GE Healthcare) with 40% formamide at 42°C overnight. Subsequently, washing was performed in 1 × SSC, 0.2% SDS for 10 min, 0.1 × SSSC, 0.2%SDS for 10 minutes, 0.1 × SSC, for 10 minutes. The arrays were then dried and imaged on GenePix^® ^Pro (Axon Instruments) at identical PMT settings. For array CGH evaluation, amplified and labelled genomic DNA were hybridized on 3 K BAC microarray platform (Perkin Elmer, US) following manufacturer recommended conditions. Signals intensities from microarray spots were quantified using GenePix Pro software (Axon Instruments) and data analyzed using Microsoft Excel^®^.

### Ozone and photo stability

Ozone resistance of fluorescent dyes was tested by exposing Cy5 and HyPer5 hybridized microarrays to 300 parts per billion (ppb) ozone generated using an ozone generator(Ozone Direct, Corona Ozone generator, Cardiff, UK). For each exposure, arrays were placed inside a closed container along with the ozone generator. Ozone generator was turned on and ozone delivered at 300 ppb for 5 minute intervals lasting up to 15 minutes (three treatments) with immediate scanning after each treatment. Since ozone tends to be unstable, high (300 ppb) doses of gas were necessary for each treatment.

Photo-stability (dye photobleaching) of Cy5 and HyPer5 was measured two ways. First, duplicate samples of both dyes were exposed to an incandescent light source for seven days, and their absorbance measured at specified times. The photostability was calculated by comparing signal from exposed samples to respective controls kept in the dark. Photostability based on array rescanning was measured by the ability of CGH arrays to be rescanned multiple times over a period of 14 days without suffering any distortion in dye ratios or loss in aberration detection.

## Competing interests

The authors declare that they have no competing interests.

## Authors' contributions

MD, TG, CC, SL participated in microarray labeling and hybridization experiments with HyPer5 and Cy3/Cy5 dyes. RR performed the photostability experiments comparing HyPer5 performance against Cy5. MC assisted in ozone and photostability design of experiments. PK conducted the ozone experiments with dyes. TV and JV carried out the array CGH experiments and analysis. All authors approved the final manuscript.
